# The role of microRNA in cancer cachexia and muscle wasting: A review article

**DOI:** 10.22088/cjim.12.2.124

**Published:** 2021-03

**Authors:** Noorwati Sutandyo

**Affiliations:** 1 Hematology and Medical Oncology Division, Dharmais National Cancer Centre Hospital

**Keywords:** MicroRNA, Cancer, Cachexia, Muscle wasting

## Abstract

Almost half of cancer patients experience cachexia syndrome. Cachexic patients are at risk of increased side effects of chemotherapy, reduced tolerance to chemotherapy drugs, longer duration of treatment period, and decreased quality of life. Cancer cachexia is a multifactorial syndrome. Micro ribonucleic acid (miRNA), a “non-coding RNA”, is considered to be a risk factor of cachexia and muscle wasting in cancer patients. miRNA has a role in affecting protein regulation, associated with different inflammatory and disease pathways. miRNA can also affect cytokines or directly change the regulation of metabolism that lead to cachexia. In this review, we want to focus on the pathophysiology to give a better understanding about the role of miRNA in the development of cancer cachexia. Based on various pathways of miRNA in cancer cachexia, it can be a potential target for therapeutic strategies. Improved knowledge about miRNA can give the opportunity to develop new treatment in the management of cancer cachexia.

Cancer is the first cause of death in the developing countries and the second cause of death in the developed countries. The new cases of cancer are estimated to reach 29.5 million by 2040 globally (1). About half of all cancer patients experience cachexia syndrome (2), a multifactorial syndrome characterized by skeletal muscle mass loss, either with or without fat loss, which is not fully reversible with the support of conventional nutrition and leads to progressive functional disorders (3). Cachexic patients are at risk of increased side effects of chemotherapy, reduced tolerance to chemotherapy drugs, longer duration of the treatment period, and decreased quality of life (4). The loss of skeletal muscle mass is proven as an independent predictor of mortality, therefore, it is important to be assessed in cancer patients (5). Skeletal muscle is the most dynamic organ in human body, accounts for approximately 40% of body weight, contains 50−75% of protein, and involved in 30−50% of protein turn-over in the body. Muscles are formed by 75% water, 20% protein, and 5% other substances, such as inorganic salts, minerals, fats, and carbohydrates. In general, muscle mass depends on protein synthesis and degradation balance, both of which are greatly influenced by several factors, such as nutritional status, hormonal balance, physical activity, exercise, injury, and disease (HIV, cancer) (6). The link between cancer and muscle wasting is generally divided into three ways, namely tumor factors (tumor-derived-factors or cytokines released by the tumor), microenvironment factors (cytokines), and host factors (surgical and chemo-radiation side effects, anorexia, dysphagia) (3). 

With the development of science, an additional mechanism of muscle wasting and cachexia in cancer patients is now found, called the micro ribonucleic acid or popularly known as “miRNA”. Decades ago, we only understood RNA as an intermediate between DNA to protein. However, diverse classes of non-coding RNAs turned out to play an essential role in gene regulation at various levels, either production, stability or translation of specific mRNA gene products (7). Previous study showed that circulating miR-206 is overexpressed in advanced stage lung cancer as well as head and neck cancer patients. When compared to normal healthy subjects, serum miR-206 expression levels were 13 times higher in lung cancer and 19, 8 times higher in head and neck cancer patients (8). In this paper, we will focus solely on the role of miRNA in the pathophysiology of cancer cachexia and muscle wasting.

**What is a micro RNA? **There are trillions of cells in the human body with different shapes and sizes. These tiny organelles are the basic units of living organisms, containing the nucleus as a central regulator of cell functions as it contains our genetic code. This process occurs through a translation process that is carried out by a combination of RNA and specific proteins in all living cells (9). RNA is divided into two classes, messenger RNA (mRNA) and non-coding RNA. Non-coding RNA can then be divided into two types, namely housekeeping non-coding RNA-- in the form of transfer RNA (tRNA) and ribosomal RNA (rRNA); and regulatory non-coding RNA. Regulatory non-coding RNA is divided into two types based on the number of constituent nucleotides, namely long regulatory non-coding RNA (more than 200 nucleotides) and short regulatory non-coding RNA (less than 200 nucleotides) (9, 10). 

MicroRNA (miRNA), one of the non-coding RNA, is a small non-coding ribonucleic acid comprises of 18–22 nucleotides that play many important roles in the regulation of biological mechanisms in the body. To date, almost two thousand precursor miRNAs (pre-miRNAs) have been detected in humans and more than 60% of protein-coding genes in miRNA target sites (8, 11). miRNA molecules have been identified in the human genome, but their specific biological functions are not widely known (12). 

The expression of miRNA is highly dependent on tissue type, disease, and metabolic status (13). Even though a non-coding RNA is not translated into protein, it has a role in affecting protein regulation, associated with different inflammatory and disease pathways. Despite the role as hallmarks of cancer, downregulation of miRNAs expression can be both oncogenes or “oncomirs” and tumor suppressors (14, 15). Moreover, miRNA is also known as a predictor of metastasis and therapeutic response (16, 17). 

Some miRNAs are found in biological body fluids, including plasma and serum, and referred to as circulating miRNAs or “c-miRNA”. “c-miRNA” is commonly transported in extracellular vesicles such as exosomes, apoptotic bodies, micro vesicles, and high-density lipoproteins to target cells and also play a role in intercellular signalling either endocrine, paracrine or autocrine (18). Many miRNAs are expressed in most tissues and cell types. However, some are tissue‐specific (mature miRNA expression is 20‐fold higher than the mean expression in other tissues) or tissue‐enriched (mature miRNA expression is higher than in other tissues but less than 20‐fold) (19, 20). Group of miRNAs that specifically expressed by skeletal muscle cells are called myomiRs. miRNAs will be secreted actively or passively by cells if there is stimulation or damage to tissue. In muscle tissue, myofiber tissue damage triggers the passive secretion of myomiRs through the plasma membrane. However, in cancer, there was also active secretion from myomiR through an intermediate extracellular vesicle (19, 20). 

**Pathophysiology: New concept: **Proinflammatory cytokines such as IL-1, IL-6, TNF-α, IFN-γ can change two metabolic pathways, namely peripheral pathway and central mediated pathway (20). Peripheral pathway involves the process of lipolysis, protein degradation, and insulin resistance involving the role of TDF (tumor-derived-factor) in the form of LMF (Lipid Mobilizing Factor) and PIF (Proteolysis Inducing Factor). Meanwhile, central mediated pathway involves the role of hypothalamus. Anorexia and catabolism are activated through suppression of the orexigenic Ghrelin-NPY/AgRP neuron and activating anorexigenic POMC/CART neuron (21). Figure 1 showed all possible pathophysiology of cachexia in cancer.

In the new concept proposed by Camargo et al. (14), there are additional tumor product, miRNA, that can affect cytokines or directly change the metabolism regulation causing cachexia. Malignant tumors can modulate the host's intragenic miRNA expression through various pathways that can be interconnected between transcription factors (TFs) and miRNAs, miRNAs and target genes, and miRNA and its host gene (22). A single miRNA can control more than one target mRNAs expressions and each mRNA may be regulated by multiple miRNAs (23). For example, study found that TP53 directly regulates 9 miRNAs (24). Other studies also showed the alteration of miRNA regulation in response to hormonal, hypoxia, dietary, and environmental changes (25-27). 

**Figure 1:**
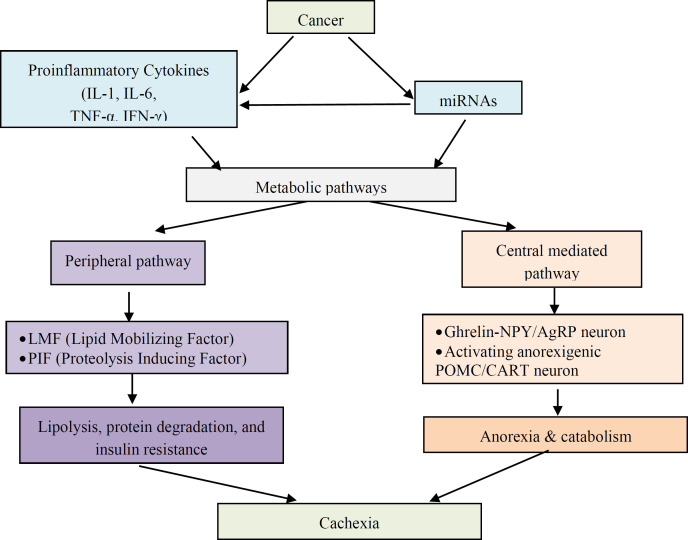
New concept of cachexia

There are many possible roles of miRNA in cancer cachexia, but this chapter will focus only on muscle-specific miRNAs (myomiR) that have been recently reported to control myogenesis (28). Subsets of miRNAs can be described as striated muscle-specific (miR-1, miR-133a, miR133-b, miR-206, miR-208, miR208b, miR486, and miR-499) or muscle‐enriched (miR-486) (20). 

The development of muscle wasting results from an imbalance between muscle protein synthesis and degradation. To understand the role of miRNA in this process, we must first understand the normal physiological process. Muscle protein synthesis is physiologically regulated in 3 stages, namely activation, proliferation, and differentiation stages. miRNA can act in all these stages (29, 30). Muscles act as an endocrine organ, which means it secretes various cytokines and peptides with paracrine and endocrine effects that are involved in various metabolic processes, referred to as myokines. One of the most studied myokines is myostatin, which is part of transforming growth factor-β (TGF-β) and plays a role in regulating the rate of basal metabolism by inhibiting the development of muscle mass (31). Other myokines that play an essential role are Pax7, Pax3 (transcription factors), and four myogenic regulatory factors (MRFs) consisting of Myf5, MyoD, MyoG, and Myf4 (32). Entering the differentiation stage, insulin-like growth factor (IGF-1) activates the PI3K/Akt signalling pathway which regulates protein degradation by ubiquitin in skeletal muscle tissue through Forkhead Box O (FOXO) signal suppression. Activated PI3K/Akt signals because increased protein synthesis through mTOR activation (33). 

The miR-486 has been shown to reduce FOXO protein expression and is a positive regulator of IGF-1/Akt pathway (30, 31). In cancer cells, miR-486 is downregulated, and the stimulation of miR-486 expression can suppress various pro-oncogenic properties and inhibit cell proliferation (34). Both of miR-206 and miR-133b are specific myomiR released only by muscle tissue. Pax7 transcription factor was a target of miR-206 as overexpression of Pax7 disturbing muscle stem cell differentiation causing muscle atrophy (35). miR-27b also found targeting Pax3 to stimulate muscle proliferation (36), while miR-133a is increased during proliferation. It reduces serum response factor (SRF), an inhibiting factor of proliferation (37). It is known that miR-1 shares high sequence homology, similar to miR-206, and both belong to miRNAs family of miR-1 (8). In bovine skeletal cell, it is proven that these miRs promote myogenic differentiation and negatively regulate Pax7 (38)*. *Several studies have also shown that miR-378 overexpression can trigger myoblast proliferation by increasing MyoD expression (39, 40). 

miR-21 has been one of mostly studied miRNA recently. It has been confirmed to activate proteolysis through toll-like receptor seven signalling, in a JNK dependent manner (32). At the late phase of differentiation, miR-181a is increased and inhibited by MyoD (40). By considering the role of miRNA in gene expression and regulation of inflammatory response especially in cancer, more information about miRNA is mandatory. Causalities of miRNA and cancer cachexia is still debatable and need more discussion. It is hoped that miRNA provides new possibilities for the development of diagnostic or prognostic biomarkers, prevention, and new target of treatment. A novel therapeutic targeting miRNAs is expected for maintaining muscle homeostasis in cancer cachexia to improve the prognosis. Further studies especially about miRNA as the target of treatment in cancer cachexia are warranted.

 In conclusion, Cancer cachexia is a complex condition associated with muscle wasting. Circulating microRNAs have recently emerged in cancer cachexia and are promising class of biomarkers. microRNAs can affect cytokines or directly change the metabolism regulation causing cachexia, one of the pathway is through muscle-specific miRNAs (myomiR) to control myogenesis. Circulating myomiRs can provide new information on skeletal muscle status and are new therapeutic avenue to maintain muscle homeostasis in cancer cachexia.

## Funding:

This article review did not receive any specific grant from funding agencies in the public, commercial or not for profit sectors.

## Disclosure:

The authors report no conflict of interest regarding this article review.
